# The Association between Blood Lipids and Systemic Lupus Erythematosus: A Two-Sample Mendelian Randomization Research

**DOI:** 10.3390/metabo13010027

**Published:** 2022-12-23

**Authors:** Yang Ding, Shasha Fan, Yi Tang, Mengjiao He, Mingyang Ren, Yunjuan Shi, Xiaohua Tao, Wei Lu

**Affiliations:** 1 Health Management Center, Department of Dermatology, Zhejiang Provincial People’s Hospital, Affiliated People’s Hospital of Hangzhou Medical College, No. 158, Shangtang Road, Hangzhou 310014, China; 2 Departments of Environmental Health, School of Public Health, Hangzhou Normal University, Hangzhou 310009, China; 3 Graduate School of Bengbu Medical College, Bengbu 233030, China

**Keywords:** blood lipids, systemic lupus erythematosus, Mendelian randomization

## Abstract

We evaluated the causal effects of blood lipid levels on systemic lupus erythematosus with a two-sample Mendelian randomization analysis. Independent single-nucleotide polymorphisms related to blood lipids levels (*p* < 5 × 10^−8^) were selected as instrumental variables (IVs) from a published genome-wide association study (GWAS). SLE GWAS analysis that included 4036 cases and 6959 controls of European ancestry provided the related roles between instrumental variables and result (SLE). The causal effects were evaluated with two-sample Mendelian randomization (MR) analyses. According to the inverse-variance weighted approaches, genes predictive of increased LDL cholesterol (OR: 1.131; 95% CI: 0.838, 1.528; *p* = 0.420), HDL cholesterol (OR: 1.093; 95% CI: 0.884, 1.352; *p* = 0.412), triglycerides (OR: 0.903; 95% CI: 0.716, 1.137; *p* = 0.384), Apolipoprotein A-I (OR: 0.854; 95% CI: 0.680, 1.074; *p* = 0.177), and Apolipoprotein B (OR: 0.933; 95% CI: 0.719, 1.211; *p* = 0.605) were not causally related to the risk of SLE, consistent with multivariate Mendelian randomization analysis. The reverse-MR analyses showed no massive causal roles between SLE and LDL cholesterol (OR: 0.998; 95% CI: 0.994, 1.001; *p* = 0.166) as well as Apolipoprotein B (OR: 0.998; 95% CI: 0.994, 1.001; *p* = 0.229). Nevertheless, a causal role of SLE in decreasing HDL cholesterol (OR: 0.993; 95% CI: 0.988, 0.997; *p* = 0.002), triglycerides (OR: 0.996; 95% CI: 0.993, 0.999; *p* = 0.010), and Apolipoprotein A-I (OR: 0.995; 95% CI: 0.990, 0.999; *p* = 0.026) was validated to some extent. Our study found no causal association between abnormal blood lipids and SLE nor a causal effect between SLE and LDL cholesterol as well as Apolipoprotein B. Nevertheless, some evidence showed that SLE exerted a causal effect on lowering HDL cholesterol, Apolipoprotein A-I, and triglyceride levels.

## 1. Introduction

As a prototypical autoimmune disease, systemic lupus erythematosus (SLE) has characteristics such as chronic inflammation and immune complex deposition in affected organs [1,2]. It has been estimated that the prevalence of SLE is 30 to 50 patients per 100,000, equating to about 500,000 patients in Europe, and 90% of SLE patients are women of childbearing age [3]. Emerging evidence suggests that the effects of genetic factors, local environments, and sex hormone metabolism contribute to SLE’s pathogenesis and progression [3,4].

The “lupus pattern” of lipoproteins in SLE has characteristics such as improved triglyceride and very-low-density lipoprotein concentrations combined with declined high-density lipoprotein (HDL) cholesterol levels, usually happening in the active stages of the disease [5]. According to a remarkable outcome, these changes are aggravated by disease activity. Growing TG levels and declining HDL cholesterol levels have been shown to be directly related to SLE disease activity index (SLEDAI) marks, indicating a lipid profile abnormality in SLE patients [6]. And the potential mechanism of SLE on HDL and Apolipoprotein A-I could be that responses such as oxidative stress and chronic inflammation among SLE patients cause changes in HDL particle size, proteomics, and lipidomics, reducing the effects mentioned above. Although blood lipids exert a great effect on SLE, the causal association between blood lipid levels and the risk of SLE is still elusive.

As a new approach for instrumental variable (IV) analysis, Mendelian randomization (MR) identifies the causative relationship between exposure and disease [7] with genetic variants including single nucleotide polymorphisms (SNPs). Since genetic variation is not affected by result or confounding status, MR methods can settle the hidden confounding effects and reverse the causality of exposure and result [8]. Furthermore, there is minimal risk of reverse causality because the disease does not influence the individual’s genotype [9]. The two-sample MR research design uses genetic data on exposure and result from large sample sizes of different populations, which improves the test’s effectiveness and provides a powerful method for estimating the hidden causal effect of the exposure on the result [10,11].

To this date, no study has attempted to elucidate whether there is a causal relationship between blood lipids and SLE risk using MR methods. Therefore, the causal relationship behind this association remains largely unclear. To that end, the published data from a lot of genetic research studies are collected to explore if blood lipids had a causal correlation with the risk for SLE with the two-sample methods for MR analysis.

## 2. Methods

### 2.1. Data Resources and Study Design

In the present study, summary statistics for low-density lipoprotein (LDL) cholesterol, triglycerides, HDL cholesterol, and Apolipoprotein A-I and Apolipoprotein B were acquired from a genome-wide association study (GWAS) from the UK Biobank (UKB) in 393,193 UKB participants of European ancestry [12]. UKB is a very large, population-based prospective cohort recruiting above 500,000 men and women between 40 and 96 years old between 2006 and 2010, and their different health-related outcomes have been followed long-term [13]. Data for SLE was obtained from a previous meta-analysis of GWAS with over 10,000 subjects of European ancestry, such as 4036 SLE cases and 6959 controls (1260 controls of mainly southern European ancestry and 5699 from the University of Michigan Health and Retirement Study), covering 644,674 markers in total [14]. All cases satisfied the standard American College of Rheumatology (ACR) classification for SLE diagnosis. Collectively, this study identified 43 susceptibility loci associated with SLE [14].

Three core assumptions for MR analysis are shown below:

(a) The genetic variant must be strongly related to the exposure [15]. Our analysis evaluated the strength of the instrument–exposure association (e.g., F statistic > 10 for the instrument–exposure association) with F statistic [16].
(b) The selected genetic variant should be related to the outcome risk only through exposure, not via confounders. Herein, the horizontal pleiotropy pathway between the genetic variant and outcome was identified with MR-Egger regression [17].
(c) The genetic variant should be independent of confounders.

### 2.2. Selection of Instrumental Genetic Variables

Our major exposure was genetically decided plasma lipids as an instrumental variable based on genetic variation related to the extents of HDL cholesterol, LDL cholesterol, triglycerides, and Apolipoprotein A-I and Apolipoprotein B at genome-wide significance levels (*p* < 5 × 10^−8^). For no bias of powerful linkage disequilibrium (LD), the SNPs related to blood lipids had to satisfy r^2^ < 0.001 thresholds and be located 10, 000 kb apart from each other [18]. For evaluating whether the SNPs were related to confounding or risk elements, potentially related traits at genome-wide significance threshold were searched for with the PhenoScanner (http://www.phenoscanner.medschl.cam.ac.uk/, accessed on 8 September 2021) [19]. Finally, we selected 112 SNPs as instrument variables for LDL cholesterol, 262 SNPs for HDL cholesterol, 213 SNPs for triglycerides, 198 SNPs for Apolipoprotein A-I, 133 SNPs for Apolipoprotein B, and 45 SNPs for SLE.

### 2.3. Statistical Analysis

**Figure 1:**
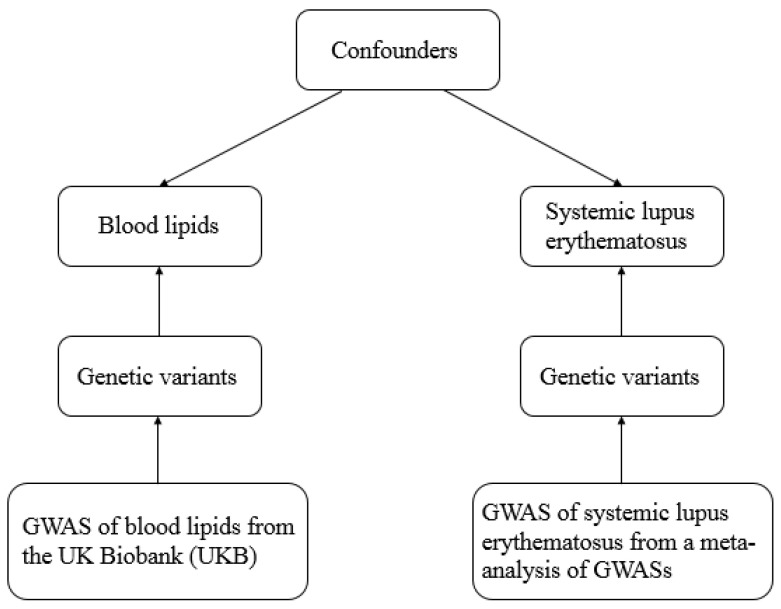
Workflow of two-sample MR for causal effect between blood lipid levels and SLE.

The potential causal associations between blood lipids and SLE in two populations, respectively (Figure 1), were evaluated with two-sample MR analysis. The correlation between blood lipids and the risk of SLE was evaluated by making the primary analyses with the inverse-variance weighted (IVW) approach. IVW approach was adopted as the primary MR analysis, requiring all selected SNPs to be effective IVs [20]. Complementary analyses, including the weighted median method [21], maximum likelihood [22], robust adjusted profile score (RAPS) [22], and MR-Egger method [21], were performed to complement IVW. In consideration of the genetic and phenotypic correlation of lipid attributes, as previously disclosed, the roles of various lipid traits in SLE were assessed with multivariable IVW and a linear regression method. Heterogeneity among the estimates from each SNP was assessed with Cochran’s Q test. A fixed-effects model was adopted when there was no statistically significant heterogeneity; otherwise, more conservative estimates were provided with the random-effects model [23].

We first assessed potential pleiotropic effects with MR-Egger regression in the sensitivity analysis. The MR-Egger regression dealt with regression dilution bias, and the mean level pleiotropic role of all genetic variants could be explained by the intercept term [17]. Moreover, we identified outlier variants for removal to rectify hidden directional horizontal pleiotropy and solve detected heterogeneity with the MR pleiotropy residual sum and outlier (MR-PRESSO) global test [24]. A leave-one-out sensitivity analysis was also made to further assess the independent validity of each IV.

Statistical significance was set as a two-tailed *p*-value < 0.05 unless otherwise noted. Furthermore, the package “Two-Sample-MR” (version 0.5.6) Auckland, New Zealand and “MR-PRESSO” (version 1.0) in R (version 4.0.5) (Auckland, New Zealand)were adopted to make all analyses.

## 3. Results

### 3.1. Genetic Instrumental Variants Selection

Using the above methods, the potential SNPs were screened for this study. Due to the confounding factors, 29 SNPs as instrument variables for LDL cholesterol, 30 SNPs for HDL cholesterol, 33 SNPs for triglycerides, 24 SNPs for Apolipoprotein A-I, and 26 SNPs for Apolipoprotein B were removed from the present study. Finally, 112, 262, 213, 198, and 133 SNPs were identified as IVs to analyze the causal effect of LDL cholesterol, triglycerides, HDL cholesterol, Apolipoprotein A-I, and Apolipoprotein B on SLE, respectively. The detailed information on the IVs, including effect allele, effect allele frequency, role sizes in blood lipids, and SLE, are displayed in Supplementary Tables S1–S5.

### 3.2. Two-Sample and Multivariable Mendelian Randomization of Blood Lipids and the Risk of SLE

IVW estimates uncovered that genetically forecasted LDL cholesterol (Odds ratio OR: 1.131; 95% confidence interval CI: 0.838, 1.528; *p* = 0.420), HDL cholesterol (OR: 1.093; 95% CI: 0.884, 1.352; *p* = 0.412), triglycerides (OR: 0.903; 95% CI: 0.716, 1.137; *p* = 0.384), Apolipoprotein A-I (OR: 0.854; 95% CI: 0.680, 1.074; *p* = 0.177), and Apolipoprotein B (OR: 0.933; 95% CI: 0.719, 1.211; *p* = 0.605) were not causally related to risk of SLE (Table 1), consistent with the results of complementary analyses (Table 1). The outcomes of MR-PRESSO are presented in Supplementary Table S11. 

In the multivariable Mendelian randomization with mutual adjustment of blood lipid values, blood lipid expression was not correlated with SLE risk. The outcome conformed to complementary analyses with a linear regression-based method (Supplementary Table S13).

### 3.3. Two-Sample Mendelian Randomization of SLE and the Risk of Blood Lipids

For examining the causal correlation between SLE and blood lipids, an MR analysis was made with SLE as the exposure and blood lipids as the outcome. There were 45 obvious SNPs (*p* < 5 × 10^−8^) related to the risk of SLE derived from the GWAS research on Bentham et al. [14]. Due to the confounding factors, four SNPs (rs6679677, rs389884, rs2736332, and rs597808) were eliminated from the current research. Thus, 41 SNPs were included in the MR analysis finally. The IVW estimate revealed no obviously causal correlation between the risk of SLE and LDL cholesterol (OR: 0.998; 95% CI: 0.994, 1.001; *p* = 0.166) and Apolipoprotein B (OR: 0.998; 95% CI: 0.994, 1.001; *p* = 0.229). However, there was a weak causal correlation between the risk of SLE and HDL cholesterol (OR: 0.993; 95% CI: 0.988, 0.997; *p* = 0.002), triglycerides (OR: 0.996; 95% CI: 0.993, 0.999; *p* = 0.010), and Apolipoprotein A-I (OR: 0.995; 95% CI: 0.990, 0.999; *p* = 0.026) (Table 2). Consistent results were also obtained in complementary analyses (Table 2). The results of MR-PRESSO are presented in Supplementary Table S12. The details of the analyses are shown in Supplementary Tables S6–S10.

## 4. Discussion

The present research found no evidence of a link between genetically forecasted blood lipid levels and the risk of SLE. Moreover, our reverse-MR analyses revealed that SLE had no significant causal effects on LDL cholesterol or Apolipoprotein B. Nevertheless, some evidence proved that SLE exerted a causal effect on lowering HDL cholesterol, Apolipoprotein A-I, and triglycerides.

Evidence supporting weak causal roles between blood lipid levels and SLE was obtained. A recent study found that HDL cholesterol levels were lower in aging *gld* mice, which was associated with the development of SLE [25]. In addition, treating these mice with lipid-free Apolipoprotein A-I reversed the autoimmune phenotype and reduced the quantity of lymphatic nodules. Therapeutic strategies using Apolipoprotein A-I and Apolipoprotein A-I-mimetic peptides have also been initiated in animal models of SLE [26,27]. Furthermore, the high potential of the ubiquitin–proteasome system in regulating many human diseases is beginning to receive broad recognition. Proteins of the ubiquitin–proteasome system and E3 ubiquitin ligases are emerging as promising molecular targets for drug discovery in various diseases [28]. In humans, decreased LDL cholesterol, Apolipoprotein A-I, Apolipoprotein B, and elevated triglycerides are often found in SLE patients, while HDL cholesterol levels are comparable to those in healthy individuals [29]. Furthermore, another study found that, in patients with SLE, HDL cholesterol and Apolipoprotein A-I levels were significantly lower, triglyceride levels were significantly higher, while increases in LDL cholesterol and Apolipoprotein B levels were not statistically significant [30]. However, inconsistent with these observational studies, no massive evidence of a causal role between blood lipid levels and increased SLE risk was observed. These conflicting outcomes could be possibly boosted by reverse causation, confounding, or selection biases inborn in conventional observational research studies. Collectively, the findings of this study indicate a complicated causal effect on blood lipids in SLE requiring further examination.

Our reverse-MR analyses showed no massive causal roles of SLE in LDL cholesterol and Apolipoprotein B. Nevertheless, several evidences provided a causal effect of SLE susceptibility on decreasing HDL cholesterol levels and Apolipoprotein A-I. In line with observational studies, massive evidence of a causal role of higher risk of SLE in individuals with lower HDL cholesterol and Apolipoprotein A-I levels was observed. The mechanism could be that responses such as oxidative stress and chronic inflammation among SLE patients cause changes in HDL particle size, proteomics, and lipidomics, reducing the effects mentioned above. In inflammatory conditions, the antioxidant effect of HDL may be altered by changed gene expression of HDL-related proteins, including amyloid A, or by changed HDL function and composition, leading to decreased Apolipoprotein A-I levels [31,32]. Furthermore, because of integrated inflammation in lupus, the decreased antioxidant capacity of HDL may decrease its anti-atherogenic role [31]. Furthermore, we found causal effect of SLE susceptibility on decreasing triglycerides levels. However, the underlying mechanism has not been reported. The effect of the SLE on triglycerides levels needs more studies to examine.

Our MR approach has several advantages. To begin, its design minimized the potential for confounding or reverse causality in observational studies. Furthermore, we tested the effect of lipids in a large cohort of SLE patients using a two-sample MR approach (4036 SLE cases and 6959 controls).

Nevertheless, the present study has several limitations. First, there was heterogeneity in our results. It was impossible to investigate any potential non-linear relationship or stratification effect that varies with age, gender, or health status, which could be a source of heterogeneity based on the GWAS data. Second, due to the weakness of the MR analysis, the second and third assumptions could not be assessed accurately, potentially leading to bias. Third, the included study subjects were of European ancestry, limiting the applicability of the findings to other study populations of different ethnicities [33]. Furthermore, we did not investigate the correlation between blood lipids and different types of SLE.

## 5. Conclusions

It is concluded that the current research did not support the causal association between blood lipid levels and SLE risk, nor did it support the correlation between SLE risk and LDL cholesterol and Apolipoprotein B levels. Nevertheless, several evidences proved a causal effect of SLE on decreasing HDL cholesterol levels, Apolipoprotein A-I, and triglycerides. As a result, more research with updated data from huge genetic research studies is needed to confirm the findings of our MR research.

## Figures and Tables

**Table 1:** Two-sample Mendelian randomization of blood lipids levels and the risk of SLE.

Exposures and Methods	SNPs	Beta	OR (95%CI)	*p* Value for Association	*p* Value for Cochran *Q* Test	*p* Value for MR-Egger Intercept
LDL cholesterol						
IVW(re)	100	0.124	1.131 (0.838, 1.528)	0.420	<0.05	
IVW(fe)	100	0.124	1.131 (0.906, 1.414)	0.277		
MR-Egger	100	0.326	1.385 (0.870, 2.204)	0.172		0.266
Weighted median	100	0.281	1.325 (0.933, 1.882)	0.116		
Maximum likelihood	100	0.125	1.133 (0.906, 1.418)	0.273		
MR.RAPS	100	0.027	1.027 (0.809, 1.305)	0.825		
HDL cholesterol						
IVW(re)	233	0.089	1.093 (0.884, 1.352)	0.412	<0.05	
IVW(fe)	233	0.089	1.093 (0.931, 1.284)	0.277		
MR-Egger	233	0.175	1.191 (0.860, 1.650)	0.294		0.497
Weighted median	233	0.094	1.099 (0.820, 1.471)	0.528		
Maximum likelihood	233	0.090	1.095 (0.931, 1.287)	0.274		
MR.RAPS	233	0.084	1.088 (0.889, 1.331)	0.412		
Triglycerides						
IVW(re)	188	−0.103	0.903 (0.716, 1.137)	0.384	<0.05	
IVW(fe)	188	−0.269	0.764 (0.634, 0.920)	0.562		
MR-Egger	188	−0.006	0.994 (0.682, 1.448)	0.974		0.525
Weighted median	188	−0.263	0.769 (0.547, 1.082)	0.131		
Maximum likelihood	188	0.269	0.764 (0.633, 0.923)	0.424		
MR.RAPS	188	−0.098	0.907 (0.724, 1.136)	0.394		
Apolipoprotein A-I						
IVW(re)	172	−0.157	0.854 (0.680, 1.074)	0.177	<0.05	
IVW(fe)	172	−0.157	0.854 (0.709, 1.030)	0.099		
MR-Egger	172	0.036	1.036 (0.714, 1.505)	0.851		0.202
Weighted median	172	0.001	1.001 (0.737, 1.359)	0.997		
Maximum likelihood	172	−0.159	0.853 (0.707, 1.030)	0.099		
MR.RAPS	172	−0.089	0.915 (0.737, 1.135)	0.419		
Apolipoprotein B						
IVW(re)	117	−0.069	0.933 (0.719, 1.211)	0.605	<0.05	
IVW(fe)	117	−0.069	0.933 (0.778, 1.120)	0.460		
MR-Egger	117	0.064	1.066 (0.746, 1.522)	0.727		0.288
Weighted median	117	−0.025	0.975 (0.715, 1.329)	0.872		
Maximum likelihood	117	−0.684	0.934(0.777, 1.122)	0.465		
MR.RAPS	117	−0.101	0.904 (0.750, 1.089)	0.290		

IVW(re), random-effects inverse-variance weighted method; IVW(fe), fixed-effects inverse-variance weighted method; RAPS, robust adjusted profile score.

**Table 2:** Two-sample Mendelian randomization of SLE and the risk of blood lipid levels.

Outcomes and Methods	SNPs	Beta	OR (95%CI)	*p* Value for Association	*p* Value for Cochran *Q* Test	*p* Value for MR-Egger Intercept
LDL cholesterol						
IVW(re)	33	−0.002	0.998 (0.994, 1.001)	0.166	<0.05	
IVW(fe)	33	−0.002	0.998 (0.995, 1.000)	0.953		
MR-Egger	33	0.002	1.002 (0.995, 1.010)	0.526		0.164
Weighted median	33	−0.001	0.999 (0.995, 1.003)	0.634		
Maximum likelihood	33	−0.002	0.998 (0.994, 1.001)	0.974		
MR.RAPS	33	−0.003	0.997 (0.993, 1.001)	0.077		
HDL cholesterol						
IVW(re)	29	−0.007	0.993 (0.988, 0.997)	0.002	<0.05	
IVW(fe)	29	−0.007	0.993 (0.990, 0.996)	0.001		
MR-Egger	29	−0.004	0.996 (0.986, 1.006)	0.471		0.466
Weighted median	29	−0.004	0.996 (0.991, 1.000)	0.060		
Maximum likelihood	29	−0.007	0.993 (0.990, 0.996)	0.001		
MR.RAPS	29	−0.006	0.994 (0.988, 1.000)	0.054		
Triglycerides						
IVW(re)	31	−0.004	0.996 (0.993, 0.999)	0.010	0.122	
IVW(fe)	31	−0.004	0.996 (0.993, 0.999)	0.003		
MR-Egger	31	−0.003	0.997 (0.991, 1.004)	0.472		0.658
Weighted median	31	−0.002	0.998 (0.994, 1.002)	0.285		
Maximum likelihood	31	−0.004	0.996 (0.993, 0.999)	0.003		
MR.RAPS	31	−0.004	0.996 (0.902, 0.909)	0.010		
Apolipoprotein A-I						
IVW(re)	25	−0.005	0.995 (0.990, 0.999)	0.026	<0.05	
IVW(fe)	25	−0.005	0.995 (0.992, 0.998)	0.001		
MR-Egger	25	−0.005	0.995 (0.983, 1.006)	0.373		0.988
Weighted median	25	−0.005	0.995 (0.990, 1.000)	0.075		
Maximum likelihood	25	−0.005	0.995 (0.991, 0.998)	0.001		
MR.RAPS	25	−0.005	0.995 (0.989, 1.000)	0.082		
Apolipoprotein B						
IVW(re)	33	−0.002	0.998 (0.994, 1.001)	0.229	<0.05	
IVW(fe)	33	−0.002	0.998 (0.995, 1.001)	0.111		
MR-Egger	33	0.004	1.004 (0.996, 1.012)	0.341		0.098
Weighted median	33	−0.003	0.997 (0.993, 1.002)	0.265		
Maximum likelihood	33	−0.002	0.998 (0.995, 1.001)	0.115		
MR.RAPS	33	−0.003	0.997 (0.993, 1.001)	0.050		

IVW(re), random-effects inverse-variance weighted method; IVW(fe), fixed-effects inverse-variance weighted method; RAPS, robust adjusted profile score.

## Data Availability

Not applicable.

## Supplementary Materials

The following supporting information can be downloaded at: https://www.mdpi.com/article/10.3390/metabo13010027/s1, Table S1. Effect estimates of genetic instrumental variables of LDL-cholesterol for SLE. Table S2. Effect estimates of genetic instrumental variables of HDL-cholesterol for SLE. Table S3. Effect estimates of genetic instrumental variables of triglycerides for SLE. Tabke S4. Effect estimates of genetic instrumental variables of Apolipoprotein A-I for SLE. Table S5. Effect estimates of genetic instrumental variables of Apolipoprotein B for SLE. Table S6. Effect estimates of genetic instrumental variables of SLE for LDL-cholesterol. Table S7. Effect estimates of genetic instrumental variables of SLE for HDL-cholesterol. Table S8. Effect estimates of genetic instrumental variables of SLE for triglycerides. Table S9. Effect estimates of genetic instrumental variables of SLE for Apolipoprotein A-I. Table S10. Effect estimates of genetic instrumental variables of SLE for Apolipoprotein B. Table S11. MR-PRESSO for causal effect between blood lipids and SLE. Table S12. MR-PRESSO for causal effect between SLE and blood lipids. Table S13. Multivariable MR for causal effect between blood lipids and SLE.
